# The effect of zinc supplementation on pro-inflammatory cytokines (TNF-α, IL-1 AND IL-6) in mice with *Escherichia coli* LPS-induced diarrhea

**Published:** 2019-10

**Authors:** Sulaiman Yusuf, Yati Soenarto, Muhammad Juffrie, Wiryatun Lestariana

**Affiliations:** 1 Department of Paediatric, School of Medicine, Universitas Syiah Kuala, Aceh, Indonesia; 2 Department of Paediatric, Dr. Zainoel Abidin Hospital, Aceh, Indonesia; 3 Department of Paediatric, School of Medicine, Gajah Mada University, Yogyakarta, Indonesia; 4 Department of Biochemistry, School of Medicine, Gajah Mada University, Yogyakarta, Indonesia

**Keywords:** Zinc, Tumor necrosis factor-α, Interleukin 1, Interleukin 6, Diarrhea

## Abstract

**Background and Objectives:**

Inflammation in the intestine causes diarrhea due to an increased release of pro-inflammatory cytokines such as TNF-α, IL-1, and IL-6. These are triggered by the exposure of *E. coli*-LPS to epithelial cells of the intestinal mucosa as well as low concentration of zinc in plasma such as in infants or children who are experiencing diarrhea. This paper aims to determine the effects of zinc supplementation on pro-inflammatory cytokines (TNF-α, IL-1 and IL-6) in mice with *E. coli*-LPS-induced diarrhea.

**Materials and Methods:**

This study used a controlled trial experimental design in the laboratory. A sample size of 20 mice were randomly divided into 4 groups: 1) Control group was given standard foods, 2) Trial group was given *E. coli*-LPS 2.5 mg/kg/oral once on day1, 3) Prevention group was given *E. coli*-LPS + 30 mg/kg/oral of zinc once daily for 12 days, 4) Therapeutic group was given *E. coli*-LPS, and were then given 30 mg/kg/oral of zinc once daily for 12 days if diarrhea occurred. Blood samples of mice were taken through the orbital sinus on the 0, 5^th^, 10^th^ hour, and on the 4^th^, 8^th^ and 12^th^ days.

**Results:**

Positive effects of zinc supplementation on levels of pro-inflammatory cytokines were observed, in which the higher levels of zinc were present, the lower levels of pro-inflammatory cytokines, especially TNF-α were observed. However, there was an increase of IL-1 and IL-6 levels on the 8^th^ day in the prevention and therapeutic groups.

**Conclusion:**

Oral zinc supplementation had a significant positive effect on the levels of pro-inflammatory cytokines. Where there were higher levels of zinc, lower levels of pro-inflammatory cytokines TNF-α were present.

## INTRODUCTION

Zinc deficiency is still common, especially in developing countries. This can be related to lack of intake, increased needs, and the loss of zinc due to diseases, especially infection. There is an association between infection and zinc deficiency which influence each other. Zinc requirements of the body will increase during infection, formation of immune functions, and new cells. Meanwhile, zinc deficiency can cause suppression of immune function, making it easier for infection to occur (1, 2).

Zinc plays a role in maintaining the integrity of the intestinal mucosa through its function in cell regeneration and membrane stability. Furthermore, Zinc has a direct impact on intestinal villi, disaccharidase brush border activity, intestinal water, and electrolyte transport. Zinc also plays a role in T cell function and enhances immunity that can reduce the severity of diarrhea (3).

*Escherichia coli*, despite being the main occupant of healthy colon flora, can also cause various diseases such as diarrhea by releasing endotoxins, which trigger the release of pro-inflammatory mediators. Lipopolysaccharide (LPS) is the main component of the cell wall of Gram-negative bacteria which is also called endotoxin and is known to be the trigger of several types of inflammatory or infectious reactions in macrophage cells and other cells that have CD14 receptors. In fact, the endotoxin (LPS) of bacteria that binds to the Toll Like Receptor (TLR) in Dendritic Cells (DC) can stimulate monocyte and macrophage cells to secrete nitric oxide (NO) and inflammatory substances (cytokines) such as Tumor Necrosis Factor-alpha (TNF-α) and interleukin-1-beta (IL 1-β), IL-6, and IL-8 (4). As a result, excessive pro-inflammatory cytokines can cause symptoms of decreased blood pressure, fever, and diarrhea (5).

In experimental studies, zinc deficiency has proven to have a direct effect on the digestive tract, by forming villous atrophy, decreasing disaccharidase enzyme activity in brush borders, and impairing intestinal transport. However, the exact mechanism that explains the pathophysiology of diarrhea due to zinc deficiency has not been agreed upon yet. Despite the uncertain mechanism, there has been proof that incidence of persistent diarrhea could be reduced by zinc supplementation and administration of oral rehydration solutions (ORS). Zinc has substantially reduced the duration and severity of diarrhea in children with acute and persistent diarrhea (6). Although the mechanism of how zinc supplementation can reduce diarrhea is still unknown, zinc therapy in diarrhea patients has shown better absorption of water and electrolytes by the intestine, faster regeneration of intestinal epithelial cells, increased levels of enzymes from the brush border, and increased immune response that can eliminates pathogens in the intestine (7). Thus, the purpose of this study was to determine the effect of zinc supplementation on pro-inflammatory cytokine (TNF-α, IL-1 and IL-6) in mice with *E. coli*-LPS -induced diarrhea.

## MATERIALS AND METHODS

This study deployed a Controlled Trial Design (8) and was conducted at The Laboratory of Nutrition and Food Centre (PSPG) at Gadjah Mada University Yogyakarta. The sample size was 20 white mice *Sprague Dawley* which were randomly chosen and randomly divided into 4 groups, with each group consisting of 5 mice. The 1^st^ group (G1) or the Control Group, was given standard nutrition. The 2^nd^ group, the Trial Group (G2), was given 2,5 mg/kg/oral of *E. coli*-LPS for three days, from day 1 to day 3. The 3^rd^ group, the Prevention Group (G3) was given 2,5 mg/kg/oral of *E. coli*-LPS on day 1 and also 30 mg/kg/oral of zinc from day 1 until Day 12, and the 4^th^ group, the Therapeutic Group (G4), was given 2,5 mg/kg/oral of *E. coli*-LPS on day 1. When diarrhea occurred, the mice in G4 were given 30 mg/kg/oral of zinc from the 1^st^ until the 12^th^ day. A 2ml sample of the mice blood was taken from sinus orbital with ELISA test technique (9) at the baseline, on the 5^th^, and 10^th^ hour, and on the 4^th^, 8^th^, and 12^th^ day to analyze zinc and pro-inflammatory cytokines (TNF-α, IL-1, IL-6) level in its serum.

## RESULTS

The characteristics of subjects in this study including weight and Hemoglobin level (Hb) are presented in Table 1.

**Table 1:** The characteristics of study groups consisted of 5 white mice

**Group**	**Mean of weight (gram)**	**Standard of deviation**	p-value^*^
G1	73,4	8,02	0,04
G2	88,4	10,92	
G3	93,8	15,06	
G4	89,6	7,335	

Group	Mean of Hemoglobin (g%)	Standard of deviation	p-value^*^

G1	11,88	0,362	0,138
G2	11,76	0,295	
G3	11,36	0,268	
G4	11,57	0,441	

* ANOVA, p < 0,05

G1= Control group G3=Prevention group G2=Trial group G4= Therapeutic group

### The level of zinc based on treatment time

**Fig. 1:**
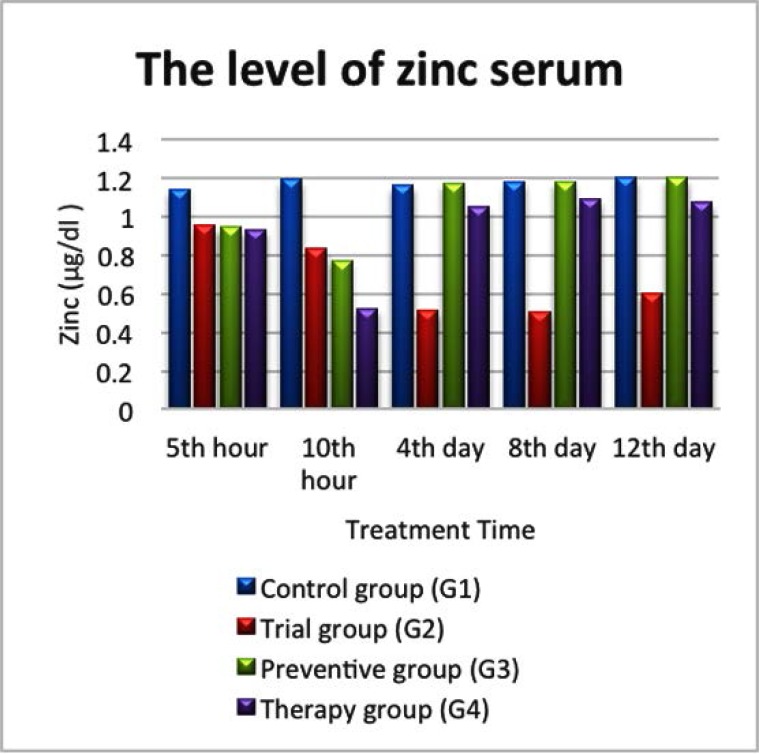
Zinc level in serum in Control (G1), Trial (G2), Prevention (G3) and Therapeutic group (G4).

The results of zinc level can be seen in Fig. 1. In the control group (G1), we observed that there was no difference in zinc level based on treatment time in 5 mice, with the level ranging from 1, 14 to 1, 2 μg/dl. However, zinc level in the trial group (G2) seemed to be lower compared to group G1, G3 and G4 which could be seen in Fig. 1. Starting from the 4^th^, 8^th^ and 12^th^ day of treatment, the 3^rd^, 4^th^ and 5^th^ mice in group G2 experienced diarrhea, respectively.

The difference could also be seen in the prevention group (G3), where zinc level increased at the 5^th^ and 10^th^ hour after being given 0,94 μg/dl and 0,77 μg/dl LPS, respectively. Meanwhile on the 4^th^, 8^th^ and 12^th^ day, zinc level was higher compared to group G2, as it can be seen in Fig. 1. This event was observed in the 3^rd^, 4^th^ and 5^th^ mice in group G3, despite that fact that all mice in this group were given zinc every day.

The same finding was observed in the therapeutic group (G4), in which zinc levels were higher in the 3^rd^, 4^th^ and 5^th^ mice compared to the mice in group G2 on the 4^th^, 8^th^ and 12^th^ day. Actually, zinc level was observed higher in the 3^rd^ mouse in G3, 3 days after receiving zinc. The same happened for the 4^th^ mouse in G4, in which zinc level was observed to be higher on the 6^th^ day after receiving zinc. Meanwhile, zinc level was higher after 10 days of receiving zinc on the 5^th^ mouse in G4, as it is shown in Fig. 1.

Zinc level in trial group (G2) was lower than control group (G1) on all the observation points (5 & 10 hours, 4, 8 and 12 days after being given LPS) as it was shown in Fig. 1. Moreover, zinc levels were also lower in the Trial group (G2) compared to the Prevention (G3) and the Therapeutic group (G4) especially on the 4^th^, 8^th^, and 12^th^ day. This might be because the mice in the Prevention and Therapeutic groups were given zinc every day, while the mice in trial group (G2) were not.

### Level of TNF-α based on treatment duration

**Fig. 2:**
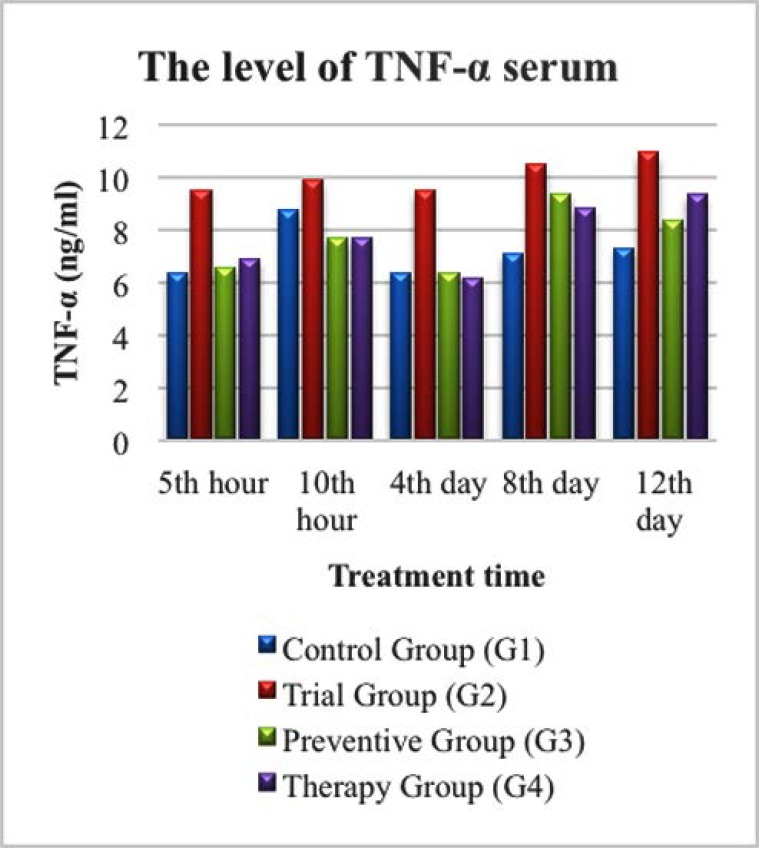
TNF-α level in serum in Control Group (G1), Trial Group (G2), Prevention Group (G3) and Therapeutic Group (G4).

The results of TNF-α levels examination were shown in Fig. 2. In the trial group (G2), a higher level of TNF-α was observed, starting from the 5^th^ & 10^th^ hour, and the 4^th^, 8^th^, and 12^th^ day compared to the other three groups (G1, G3, and G4). The TNF-α levels in G3 and G4 were noticeably lower than in G2 on the 4^th^ day. The TNF-α levels in G3 and G4 became higher on 8^th^, and 12^th^ day, but were still not as high as in G2. This finding was interesting because zinc was administered daily for mice in G3 and G4, but not for mice in G2.

The TNF-α level in G2 was higher at the 5^th^ hour after being given LPS compared to G1, G3, and G4. The TNF-α level was higher at the 10^th^ hour after being given LPS in G3, and G4 compared to the level on the 5^th^ hour in respective groups, as shown in Fig. 2.

### The level of IL-1 based on treatment duration

**Fig. 3:**
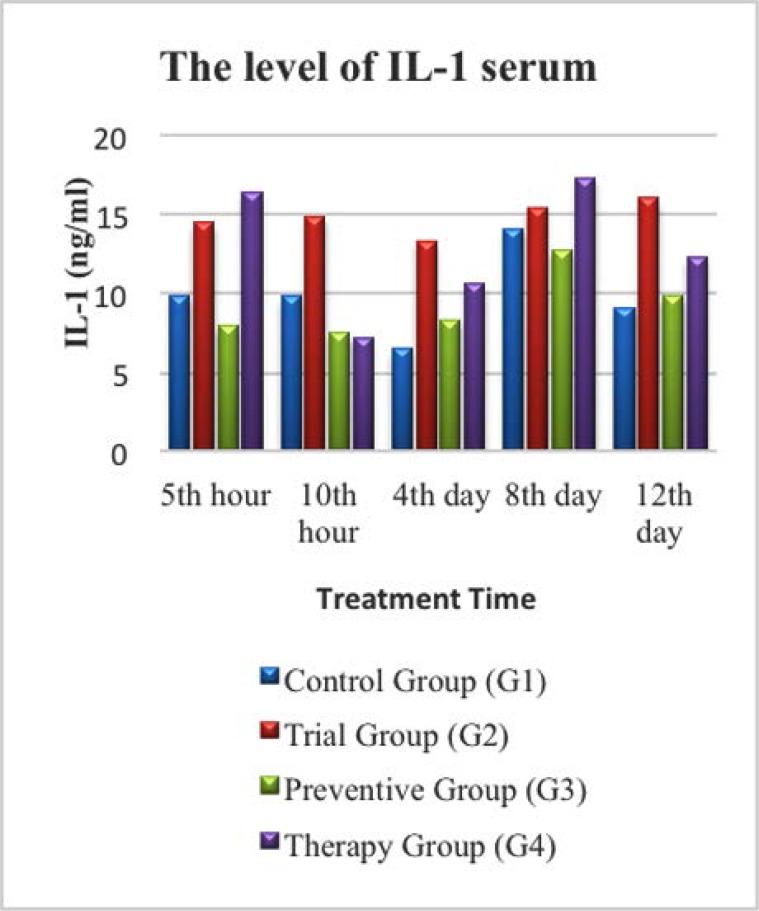
Diagram of IL-1 level in serum on control group (G1), Trial group (G2), Preventive Group (G3) and Therapy group (G4).

In this study the level of IL-1 in the control group (G1) was found to be higher on the 8^th^ day of mice (G1) 4 compared to mice (G1) 1, (G1) 2, (G1) 3, and (G1) 5, however, diarrhea did not occur in all mice in this group. In the trial group (G2), IL-1 levels were higher at the 5^th^ and 10^th^ hour after being given LPS before it went lower on the 4^th^ day in mice (G2) 3. However, subsequently higher IL-1 levels were observed on 8^th^ and 12^th^ day in mice (G2) 4 and (G2) 5, as shown in Fig. 3. In this group, three out of five mice (mice (G2) 3, (G2) 4, and (G2) 5) started to have diarrhea from the 3^rd^ day after given LPS.

From Fig. 3. it could also be seen that IL-1 levels in the therapy group (G4) were the highest among all the groups (16.33 ng/ml) at the 5^th^ hour after given LPS, but then declined to its lowest level at the 10^th^ hour. Interestingly, the IL-1 level then increased again on the 4^th^ and 8^th^ day (its peak) to just decline again on the 12^th^ day (12.30 ng/ml only).

### The level of IL-6 based on treatment duration

**Fig. 4:**
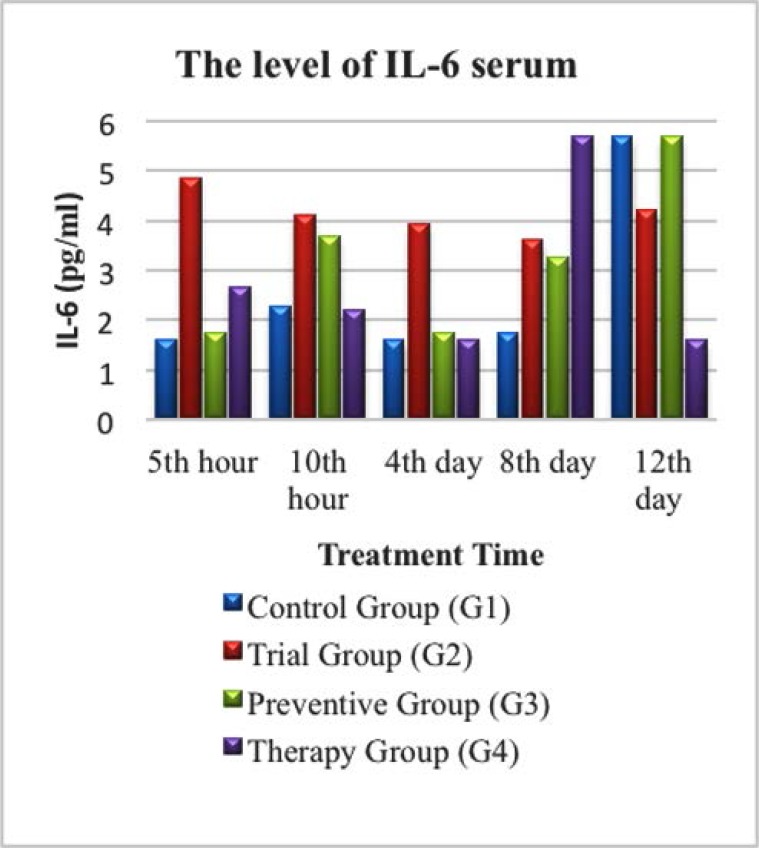
Diagram of IL-6 level in serum in control group (G1), Trial group (G2), Prevention Group (G3) and Therapeutic group (G4).

The IL-6 level in this study was observed and is shown in Fig. 4. In the control group (G1) of the 5 experimental mice, a very high IL-6 level (5.70 pg/ml) was observed on the 12^th^ day in mouse (G1) 5, although the mouse did not experience diarrhea. However in the trial group (G2), IL-6 level was higher at the 5^th^ and 10^th^ hour after given LPS compared to the control group (G1), which was 4.86 pg/ml and 4.10 pg/ml respectively. However, the IL-6 level in this group (G2) decreased on the 4^th^ and 8^th^ day in mouse (G2) 3 and (G2) 4, further higher than 4.22 pg/ml on the 12^th^ day of mice (G2) 5, shown in Fig. 4.

As shown in Fig. 4., the IL-6 level in the prevention group (G3) showed an increase at the 5^th^ hour after being given LPS. The level of IL-6 were almost the same as IL-6 levels in the control group (G1) which was 1.74 pg/ml, higher than twice from the previous value at the 10^th^ hour after treatment from mice (G3) 2. Meanwhile, in the therapeutic group (G4), the level of IL-6 increased 5 hours after treatment (2.67 pg/ml), but then declined again down to 1.60 pg / ml on the 4^th^ day from mice (G4) 3.

## DISCUSSION

### Pro-inflammatory cytokine TNF-α, IL-1 and IL-6

Existing literatures mention that the expression of pro-inflammatory cytokines increase 90 minutes after infection, reach their peak in 3 hours and return to normal 6 hours after infection. Meanwhile, the secretion of pro-inflammatory cytokines reach its maximum level in 4–6 hours after infection and return to their normal level 12 hours later (10). In this study, TNF-α level in trial group (G2) was the highest at every measurement point. Despite a small decrease on the 4^th^ day, its level was still the highest among all groups as shown in Fig. 2. Furthermore, the IL-1 level in trial group (G2) was also observed to be higher compared to the control group (G1) and the prevention group (G3). Although the IL-1 level in Group 2 was higher than in the therapeutic group (G4) at the 10^th^ hour, 4^th^ day, and 12^th^ day, however, the level of IL-1 in G4 was significantly higher than in G2 on the 5^th^ hour, and 8^th^ day as seen in Fig. 3. Although zinc can influence the production and signalling of numerous inflammatory cytokines in various cell types, Zinc supplementation is not always found beneficial. Adding zinc at very high concentrations (>100 μM) has been suggested to increase cytokine production in some cell types, as has been observed in human peripheral blood mononuclear cells (PB-MCs) harvested from healthy adults and in human promonocytic HL-CZ cells (11).

In this study, the levels of TNF-α, IL-1, and IL-6 were seen higher in the trial group (G2) compared to the control group (G1) as seen in Figs. 1–3. The levels of IL-1 and IL-6 were particularly high on the 8^th^ day in all experimental groups. Zinc deficiency, as occurred in G2, causes decreased cellular immunity which will induce the inflammatory process, characterized by an increase in the expression of pro-inflammatory cytokines in response to the exposure reaction of an antigen, as shown in this study. Free intracellular zinc acts as a suppressor of cytokine production by stimulating the immune cells. In contrast, zinc positively regulates the expression of cytokines. Zinc supplementation enhances cytokine production in immune cells. These conflicting effects may be due to the concentration-dependent effect of zinc on different signalling molecules involved in cytokine expression (12).

Tumor Necrosis Factor (TNF) is the main cytokine in the acute inflammatory response to Gram-negative bacteria and other microbes. Severe infection can trigger production of large amounts of TNF which can cause systemic reactions (4). According to Prasad, 2008, there was an increase in the release of pro-inflammatory cytokines such as TNF-α and IL-1β by monocytes and macrophages which were activated by the inflammatory process and were associated with decreased zinc status in patients. This is in accordance with the finding in this study, which showed an increase in TNF-α levels in the trial group (G2) compared to the control group (G1) seen in 2 (13). In the condition of diarrhea, zinc deficiency occurs and might reduce the production and activity of SOD enzymes, subsequently increasing the activity of free radicals that can also cause inflammatory reactions in the intestinal mucosa, which would trigger an increase of TNF-α by competent immune cells. This condition is not ideal, as a high concentration of TNF-α will damage the tight junction in enterocyte cells of the intestinal mucosa (14).

Increased levels of IL-1 and IL-6 in the diarrhea group compared to the control group in this study were due to the presence of microbes that release endotoxins and/or exotoxins, both of which promote the release of pro-inflammatory mediators. LPS is a component of the cell wall of Gram-negative bacteria, an immune system polyclonal activator, which promotes the release of various pro-inflammatory cytokines such as IL-1, IL-6, IL-12, IL-18, TNF-α, and TNF-β. Furthermore, bacterial toxins cause damage on the tissues and promote the release of thrombin, histamine, and cytokines, which can damage nerve endings (15).

### The effect of zinc supplementation on pro-inflammatory cytokines (TNF-α, IL-1 and IL-6) levels

In this study, there was an association between giving zinc supplementation and the increase of pro-inflammatory cytokine levels. The higher zinc level is, the lower TNF-α pro-inflammatory cytokines level will be. This association was observed on 4^th^, 8^th^, and 12^th^ days. However, it was interesting to see how IL-1 and IL-6 increased on the 8^th^ day. In addition, there was also a relationship between the levels of pro-inflammatory cytokines and the incidence of diarrhea in mice as seen in Figs. 1–3.

During the inflammatory state, white adipose tissue produces cytokines such as (interleukin-6) IL-6, which stimulates the secretion of C-reactive protein (CRP) in the liver, which is a sensitive marker of inflammation, tissue damage, and impairs vascular endothelial function. On the other hand, TNF-α contributes to the acute-phase response by enabling IL-6, which increases pro-inflammatory cytokines such as adiponectin (16).

The effect of zinc supplementation on improving patients with diarrhea can be explained through the effect of zinc in inhibiting the formation of free radicals by increasing the formation of SOD, which acts as the main antioxidant enzyme that controls superoxide anions inhibiting the apoptosis process in intestinal mucosal epithelial cells. Furthermore, zinc also increases the formation of ADP ribosyl enzymes, DNA, and RNA polymerase, which all play an important role in the process of cell respiration and regeneration that stops the apoptosis process (14).

The presence of an immune response in zinc deficiency is proven through an increase of TNF-α and IL-6 level before intervention and conversely, the reduction in the immune response is characterized by a decrease in TNF-α and IL-6 level after zinc administration. This once again suggests the association mentioned before: the higher the levels of zinc, the lower of pro-inflammatory cytokines levels will be. Zinc can reduce serum cytokine levels, which means it can also control the immune response to free radicals, a process made possible by its ability to inhibit the formation of free radicals. The occurrence of intestinal mucosal inflammation is proven by the presence of faecal TNF-α and based on the role of TNF-α. It is suggested that TNF-α is responsible for damage to the intestinal mucosa because this cytokine is pleotropic, meaning it can stimulate inflammation and signal death/cell apoptosis (17).

Zinc deficiency has been associated with increase in inflammatory cytokines and other markers of inflammation. However, during the time this study was carried out, the precise mechanisms that explain how zinc deficiency contributes to increased inflammation remains a topic that can be clarified through further study (18).

Zinc is also able to stimulate the production of tumor necrosis factor-alpha (TNF-α) by monocyte cells, so that the ability of phagocytosis increases. TNF-α is a mediator for non-specific immune responses and belongs to the cytokine group. Helge and Rink reported that in vitro mononuclear cell incubation in the zinc medium can increase the production of interleukin 1, interleukin 6, tumor necrosis factor (TNF), IL-2R, and interferon (19).

Monocytes in deficient mice fail to kill intracellular parasites. The research conducted by Bires reported that phagocytic activity increased after the administration of zinc, showing an increase in the number of monocytes by 14% and granulocytes by 86% (20). Furthermore, Linder also suggested that insufficiency or excess zinc minerals can cause damage to the immune system components (21).

Cytokines are directly involved in regulating permeability and ion response in the epithelium and TNF-α has a direct effect on almost all enterocyte cell functions including transportation, permeability, regeneration, and proliferation. With the administration of zinc, inflammation of the intestinal mucosa can be reduced as proven by the decrease in serum TNF-α levels by inhibiting the production of TNF-α and IL-6. TNF-α plays an important role in the mechanism of diarrhea in zinc deficiency, while serum TNF-α level can be used as a sign of inflammation that triggers the production of cytokines (22,23).

In conclusion, zinc supplementation has a positive effect on reducing pro-inflammatory cytokines level. The higher level of zinc is, the lower level of pro-inflammatory cytokines will be, particularly for TNF-α. However, it is important to notice the increase of IL-1 and IL-6 levels on the 8^th^ day in the prevention and therapeutic groups in mice with *E. coli*-LPS -induced diarrhea.

## References

[1] Baqui HA Black RE Walker CLF Arifeen S Zaman K Zinc supplementation and serum zinc during diarrhea. Indian J Pediatr 2006; 73: 493– 497. 16816510 10.1007/BF02759893

[2] King JC ( 2003). Specific nutrient requirements. In: Nutrition And Immunology Principles And Practice. Humana Press Inc. New Jersey, USA, pp. 65– 73.

[3] Roy SK Behrens RH Haider R Akramuzzaman SM Mahalanabis D Wahed MA Impact of zinc supplementation on intestinal permeability in Bangladeshi children with acute diarrhea and persistent diarrhea syndrome. J Pediatr Gastroenterol Nutr 1992; 15: 289– 296. 1432467 10.1097/00005176-199210000-00010

[4] Kikkawa I Saito S Tominaga K Hoshino Y Ooi Y Nakano M Lippopolysaccharide (LPS) stimulates the production of tumor necrosis factor (TNF)-alpha and expression of inducible nitric oxide synthase (iNOS) by osteoclasts (OCL) in muribe bone marrow cell culture. Microbiol Immunol 1998; 42: 591– 598. 9802559 10.1111/j.1348-0421.1998.tb02329.x

[5] Baratawidjaja KG Rengganis I ( 2009). Cytokines in Basic Immunology. University of Indonesia Press, 8th ed Jakarta, Indonesia, pp. 219– 55.

[6] Bhandari N Bahl R Taneja S Strand T Molbak K Ulvik RJ Substantial reduction in severe diarrheal morbidity by daily zinc supplementation in young North Indian children. Pediatrics 2002; 109( 6): e86. 12042580 10.1542/peds.109.6.e86

[7] Fenwick PK Aggett PJ Macdonald DC Huber C Wakelin D Zinc deprivation and zinc repletion: effect on the response of rats to infection with strongyloides ratti. Am J Clin Nutr 1990; 52: 173– 177. 2360547 10.1093/ajcn/52.1.173

[8] Madiono B Moeslichan S Sastroasmoro S Budiman I Purwanto SH ( 2002). Sample Size Estimation in Basic Clinical Research Methods. Sagung Seto Press, 2nd ed Jakarta, Indonesia, pp. 259– 87.

[9] Moore KW O’Garra A de Waal Malefyt R Vieira P Mosmann TR Interleukin-10. Annu Rev Immunol 1993; 11: 165– 190. 8386517 10.1146/annurev.iy.11.040193.001121

[10] Sturniolo GC The many functions of zink in inflammatory conditions of the gastrointestinal tract. Trace Elem Exp Med 2000; 13: 33– 39.

[11] Gammoh NZ Rin Lothar Zinc in infection and inflammation. Nutrients 2017; 9( 6): E624. 28629136 10.3390/nu9060624 PMC5490603

[12] Nishida K Uchid Ryota Role of zinc signaling in the regulation of mast cell, Basophil, and T cell-mediated allergic responses. J Immunol Res 2018; 2018: 5749120. 30596108 10.1155/2018/5749120 PMC6286780

[13] Prasad AS Zinc in human health: Effect of Zinc on immune cells. Mol Med 2008, 14: 353– 357. 18385818 10.2119/2008-00033.Prasad PMC2277319

[14] Shankar AH Prasad AS Zinc and immune function: the biological basis of altered resistance to infection. Am J Clin Nutr 1998, 68( 2 Suppl): 447S– 463S. 9701160 10.1093/ajcn/68.2.447S

[15] Janeway CA Travers P Walport M Shlomchik M ( 2005). Innate Immunity. In: Immunobiology: The Immune System in Health & Disease. Garland Science Publishing, 6th ed New York, USA, pp.

[16] Olechnowicz J Tinkov A Skalny A Suliburska J Zinc status is associated with inflammation, oxidative stress, lipid, and glucose metabolism. J Physiol Sci 2018; 68: 19– 31. 28965330 10.1007/s12576-017-0571-7 PMC5754376

[17] Rosalina I ( 2007), The efficacy of Zinc administration on diarrhea, in the Proceeding of The 3rd National Meeting of Indonesian Pediatric Gastroenterology Coordination Board: A Comprehensive Management of Gastroenterohepatic Problems in Children. BKGAI, 1st ed Surabaya, Indonesia, pp. 159– 67.

[18] Wong CP Nicole AR Ho Emily Zinc deficiency enhanced inflammatory response by increasing immune cell activation and inducing IL6 promoter demethylation. Mol Nutr Food Res 2015; 59: 991– 999. 25656040 10.1002/mnfr.201400761 PMC4425307

[19] Helge K Rink L Zink-altered immune function. J Nutr 2003; 133( 5 Suppl 1): 1452S– 1456S. 12730441 10.1093/jn/133.5.1452S

[20] Bires J Bajova V Vrzgula L Strojny L Kovarova E Levkutova M Humoral immunity response to zinc injectible zindep inj a.u.v. biotika in pregnant cows. Biopharm 1991; 1: 103– 109.

[21] Linder M.C ( 1992) Nutritional Biochemistry and Metabolism. Translated version. Penerbit Universitas Indonesia, Jakarta, Indonesia.

[22] Aggarwal R Sentz J Miler MA Role of zink administration in prevention of childhood diarrhea and respiratory illness: a meta analysis. Pediatrics 2007; 119; 1120– 1130. 17545379 10.1542/peds.2006-3481

[23] Subowo ( 1993): Cytokines in Immunology. Angkasa Press, 1st ed. Bandung, Indonesia, pp. 187– 206.

[24] Scrimshaw NS SanGiovanni JP Synergism of nutrition, infection, and immunity: an overview. Am J Clin Nutr 1997; 66: 464S– 477S. 9250134 10.1093/ajcn/66.2.464S

